# Three-year trajectories of insomnia symptoms among young breast cancer survivors

**DOI:** 10.3389/fonc.2026.1967396

**Published:** 2026-10-19

**Authors:** Chloe M. Hery, Kayla Williams, Sarah A. Janse, Juan Peng, Faiza Kalam, Kimberly Van Zee, Elizabeth Z. Naftalis, Electra D. Paskett, Michelle J. Naughton

**Affiliations:** 1 Division of Cancer Prevention & Control, College of Medicine, The Ohio State University, Columbus, OH, United States; 2 The Ohio State University Comprehensive Cancer Center, Columbus, OH, United States; 3 Center for Biostatistics, Department of Biomedical Informatics, The Ohio State University, Columbus, OH, United States; 4 Breast Service, Department of Surgery, Memorial Sloan Kettering Cancer Center, New York, NY, United States; 5 Health Texas Community Health Services Corporate Director of Breast Services, Dallas, TX, United States

**Keywords:** breast cancer, insomnia symptoms, sleep, survivors, trajectories

## Abstract

**Background:**

Sleep disruptions are common in cancer survivors and can negatively impact quality of life. Young breast cancer survivors face unique challenges and may have distinct sleep patterns. We examined trajectories of insomnia symptoms over 3 years among young survivors.

**Methods:**

836 women, ≤45 years old with stage I-III breast cancer, were recruited to the Menstrual Cycle Maintenance and Quality of Life After Breast Cancer Study within 8 months of diagnosis. Insomnia symptoms were assessed at 6-month intervals from baseline through 3 years post-recruitment, using the Women’s Health Initiative Insomnia Rating Scale (WHIIRS). Group-based trajectory modeling was used to identify distinct trajectories of insomnia symptoms. The final model was chosen by comparing the average posterior probability of assignment (PPA) and examining the distinctiveness and interpretability of the trajectories. Key demographic, quality of life and clinical variables were assessed in multinomial logistic regression models as potential factors associated with group membership.

**Results:**

The mean age of participants was 38 years (range: 20–45 years) at enrollment. Three distinct insomnia trajectories were identified: low probability of insomnia (n=277, 33.1%), moderate probability (n=354, 42.4%), and high probability (n=205, 24.5%). Average PPA was between 75%-82% for each group. Insomnia symptoms were highest at baseline but improved for the low probability and moderate probability groups. In adjusted models, older age (p=0.02), lower education (p=0.03), having received chemotherapy (p=0.03), night sweats (p=0.02), lower SF-12 physical component scores (poorer physical functioning, p<0.0001), and lower SF-12 mental health component scores (p<0.0001) were associated with participants in the moderate and high probability insomnia groups compared to those in the low probability group.

**Conclusion:**

Insomnia symptoms are problematic for many young breast cancer survivors. Future studies are needed to determine whether early identification and intervention can modify these insomnia symptom trajectories and improve broader survivorship outcomes.

## Introduction

Breast cancer is one of the most commonly diagnosed cancers in women (1). In 2025, there were over 310,000 new invasive breast cancer diagnoses in the United States (U.S.) (1, 2). Due to increasing early detection and advances in treatment, today there are more than 4 million breast cancer survivors living in the U.S (2–4). In general, around 75% of cancer survivors experience some type of sleep disturbance (5). These sleep issues may be due to the cancer itself, side effects from treatments, or the emotional impact that cancer has on an individual (5). Specifically, common sleep issues experienced among cancer survivors often include short sleep duration, long sleep-onset latency, and frequent night awakenings (5). Sleep issues can cause problems like daytime fatigue, low energy, and reduced cognitive function (5). Sleep issues are highly prevalent in breast cancer and side effects from cancer treatment may worsen pre-existing sleep issues (6). Sleep changes in women with breast cancer may begin during initial treatment with chemotherapy or radiation therapy and can persist over the long-term in survivorship (7). Sleep problems greatly affect the quality of life of breast cancer survivors, as poor sleep quality can worsen other symptoms such as hot flashes, fatigue, pain, and depression (8, 9).

While sleep disturbances are common among all breast cancer survivors, younger women face unique additional challenges. Currently, about 10% of breast cancer patients are diagnosed under the age of 45 (10, 11). Young women with breast cancer have been reported to have worse sleep health than older women with breast cancer (12). They are also more likely to be diagnosed with more aggressive forms of breast cancer compared to older women (13). Young breast cancer survivors may also report greater psychological distress, changes in quality of life, and more physical symptoms than their older counterparts (14). These survivors are also at an even higher risk of sleep issues due to life demands, cancer treatment, future fertility concerns, and the stressors that come with a cancer diagnosis (15–17). Younger women tend to experience stress from performing multiple roles throughout their day, including being a partner, parent, and/or employee simultaneously (18). In a recent review, it was found that young breast cancer survivors have various competing demands due to their younger age and family status (19). They also experience stress from the side effects of cancer treatment, such as premature menopause, which can lead to symptoms such as hot flashes and night sweats, which also negatively impact sleep (20, 21).

Sleep health is important to maintaining physical, psychological, and social well-being. In general, poor sleep health has been shown to increase the risk of cardiovascular disease (22) and other adverse health effects, such as obesity, diabetes, and impaired mental health (23). Poor sleep quality can also negatively impact immune function, physiological and psychological functions, depression, and can increase risk for chronic fatigue (24). Sleep issues can also lead to a decline in cognitive function in breast cancer patients (9). High quality sleep is especially important when in cancer recovery. For patients who undergo surgery, there is a bidirectional relationship between sleep issues and pain, where sleep issues contribute to increased pain, and those with pain having increased sleep issues (25). Poor sleep quality has also shown to negatively impact the patients quality of life (6).

Sleep disruptions are very common in this population with sleep problems affecting 67-90% of survivors (5, 26). Most previous research examined the average effect of sleep, but does not consider distinct groups, and often has short follow-up (≤12 months). Young breast cancer survivors face unique challenges and may have distinct sleep patterns, yet many studies do not include younger survivors. Identifying subgroups with persistent versus improving insomnia symptoms may provide clinically useful information that is not captured by the average longitudinal trend and may help identify survivors who warrant closer monitoring or earlier intervention. Thus, the aim of this study was to examine distinct trajectories of insomnia symptoms over 3 years in a cohort of young breast cancer survivors and to determine sociodemographic, clinical, and quality of life factors associated with trajectory group membership.

## Methods

The Menstrual Cycle Maintenance and Quality of Life After Breast Cancer Treatment Study was a multi-center, longitudinal observational study (27). Recruitment occurred from January 1998 to November 2005 from five sites located at the Memorial Sloan-Kettering Cancer Center in New York City, New York; M.D. Anderson Cancer Center in Houston, Texas; University of Texas –Southwestern, Dallas, Texas; Presbyterian Hospital in Dallas, Texas; and the Wake Forest University Baptist Medical Center in Winston-Salem, North Carolina. Female patients who were between the ages of 18 and 45 years and had been diagnosed with stage I-III invasive breast cancer within the previous 8 months were enrolled. Participants were also required to have had regular menstrual cycles at the time of diagnosis in order to examine primary study aims regarding menstrual cycling, fertility, and menopause post-breast cancer treatment. Thus, women who had a previous hysterectomy were ineligible for this protocol. Participants were also excluded if they had any prior or concurrent history of any cancer, excluding basal or squamous cell skin carcinoma and stage 0 cervical cancer. Participants were identified soon after diagnosis using tumor or surgical registries and patient or physician referrals.

Baseline forms were completed at the time of study recruitment at the participating sites. Follow-up data collection was conducted via mail through the study coordinating center at the Wake Forest University School of Medicine. Written informed consent was obtained from all individual participants. This study was approved by the Institutional Review Board of each participating site and the U.S. Army Medical Research and Materiel Command Human Subjects’ Committee.

### Measures

#### Demographics

Information was collected during study enrollment on age, marital status, race/ethnicity, educational background, income, and employment status.

#### Insomnia symptoms outcome

Insomnia symptoms were assessed at 6-month intervals from baseline through 3 years post-recruitment, using the Women’s Health Initiative Insomnia Rating Scale (WHIIRS), a self-reported measure of insomnia symptoms (28, 29). The WHIIRS consists of five questions asking about sleep in last four weeks. Responses are summed to create a total score ranging from 0–20 points, with higher score indicating greater insomnia symptom severity. Consistent with prior research, a WHIIRS score of ≥ 9 points was used to identify participants with elevated insomnia symptoms and high likelihood of clinically significant insomnia symptoms. Participants were categorized as having “low insomnia symptoms” (score <9) and “high insomnia symptoms” (score ≥ 9).

#### Cancer clinical factors

Each recruiting institution performed extensive medical chart reviews. Information was collected on the participants’ height and weight for body mass index (BMI) calculation, the date of breast cancer diagnosis, tumor size, location, grade, estrogen receptor (ER) status (positive or negative), progesterone receptor (PR) status (positive or negative), number of positive lymph nodes, and type of definitive cancer surgery. Treatment information including chemotherapy (yes/no), radiation (yes/no) and endocrine therapy (yes/no) were also gathered from medical oncology office records.

#### Other covariates

Other pertinent variables to this analysis were reports of hot flashes and night sweats. Participants were asked if they had experienced either in the past month, and if so, to report how bothersome the symptoms were: (did not occur, mild, moderate, or severe). Health-related quality of life was assessed using the Short Form Health Survey Questionnaire (SF-12) (30) which resulted in a calculated physical component score and mental component score.

### Statistical analysis

Participant characteristics were described using frequencies, percents or means, with standard deviations depending on the variable type, overall and by insomnia trajectory membership. Chi-square tests were used to determine significant differences by insomnia trajectory.

Group-based trajectory modeling (GBTM) was used to identify distinct longitudinal trajectories of insomnia symptoms over the 36-month follow-up period (31). At each assessment, “high insomnia symptoms” was defined as sleep score ≥9 and coded as a binary outcome. Logistic GBTM was then used to model the probability of insomnia over time. Models with two to four trajectory groups and varying polynomial orders were examined. Model selection considered the Bayesian information criterion (BIC), statistical significance of polynomial terms, average posterior probabilities of assignment (PPA), trajectory group size, and the distinctiveness and interpretability of the trajectories (**Supplementary Table 1**). The selected three-group model included quadratic terms for the low- and moderate- probability trajectories and an intercept-only term for the high-probability trajectory and had a BIC of -2678.90. Each participant was assigned to the single trajectory for which they had the highest posterior probability of membership.

A candidate set of baseline demographic, clinical, treatment-related, symptom, and quality-of-life variables potentially associated with insomnia trajectory membership was identified based on clinical knowledge and the study context. Univariate analyses were used as an initial screening step, with variables demonstrating an association of p<0.1 considered for inclusion in the multivariable model. The candidate set was subsequently reviewed by the clinical investigator, and variables considered clinically important were retained regardless of their univariate statistical significance. To assess the potential impact of uncertainty in trajectory classification on the multinomial regression results, a sensitivity analysis was conducted using posterior probability-based pseudo class assignments. For each participant, trajectory membership was randomly drawn according to the estimated posterior probabilities of membership in the three trajectory groups. This procedure was repeated 500 times, the multivariable multinomial logistic model was fit to each pseudo class assignment, and parameters estimates were pooled across the 500 analyses. GBTM was conducted in STATA using the ‘traj’ plugin (31). All other analyses were performed in SAS 9.4 (SAS Institute, Cary, NC).

## Results

The average age of participants was 38 years (range: 20–45 years) at study enrollment. Most were non-Hispanic White (88.6%), had at least a 4-year college degree (67.6%), were employed full-time (49.6%) or part-time (14.3%), and were married/partnered (74.5%) (**Table 1**). The majority of cancers were stage 1 (41.2%) or stage 2 (52.1%) and there was almost an even split between surgery type, with 50.5% having a lumpectomy and 49.5% having a mastectomy. Almost all participants received chemotherapy (n=737, 88.2%), 30.5% received radiation therapy, and 59.6% received hormone therapy.

**Table 1:** Baseline demographic and clinical characteristics overall and by trajectory group membership (N = 836).

	Total (N=836)	Probability of insomnia group membership			p-value
		Low probability (N=277)	Moderate probability (N=354)	High probability (N=205)	
Age at Diagnosis					0.033
<30 years	56 (6.7%)	23 (41.07%)	24 (42.86%)	9 (16.07%)	
30 – 34.9 years	152 (18.2%)	48 (31.58%)	71 (46.71%)	33 (21.71%)	
35 – 39.9 years	249 (29.8%)	87 (34.94%)	113 (45.38%)	49 (19.68%)	
40 – 45 years	378 (45.3%)	118 (31.22%)	146 (38.62%)	114 (30.16%)	
Race					0.87
Non-Hispanic White	740 (88.6%)	239 (32.3%)	315 (42.57%)	186 (25.14%)	
Black/ African American	41 (4.9%)	16 (39.02%)	18 (43.9%)	7 (17.07%)	
Hispanic	31 (3.7%)	12 (38.71%)	12 (38.71%)	7 (22.58%)	
Asian/Pacific Islander	23 (2.8%)	9 (39.13%)	9 (39.13%)	5 (21.74%)	
Education					0.031
High school or less	74 (8.9%)	29 (39.19%)	28 (37.84%)	17 (22.97%)	
Some college	196 (23.5%)	49 (25%)	87 (44.39%)	60 (30.61%)	
College Degree	331 (39.6%)	115 (34.74%)	150 (45.32%)	66 (19.94%)	
Post-graduate	234 (28.0%)	83 (35.47%)	89 (38.03%)	62 (26.5%)	
Income					0.48
< $50,000	225 (27.4%)	67 (29.78%)	97 (43.11%)	61 (27.11%)	
$50,000-$74,999	164 (20.0%)	55 (33.54%)	65 (39.63%)	44 (26.83%)	
$75,000-$100,000	136 (16.6%)	45 (33.09%)	57 (41.91%)	34 (25%)	
> $100,000	296 (36.1%)	106 (35.81%)	131 (44.26%)	59 (19.93%)	
Employment					0.23
Full-time	414 (49.6%)	151 (36.47%)	160 (38.65%)	103 (24.88%)	
Part-time	119 (14.3%)	37 (31.09%)	58 (48.74%)	24 (20.17%)	
Other	151 (18.1%)	40 (26.49%)	70 (46.36%)	41 (27.15%)	
Homemaker	151 (18.1%)	48 (31.79%)	66 (43.71%)	37 (24.5%)	
Marital Status					0.77
Married/Partnered	623 (74.5%)	206 (33.07%)	266 (42.7%)	151 (24.24%)	
Divorced/Separated	79 (9.5%)	23 (29.11%)	37 (46.84%)	19 (24.05%)	
Never Married	134 (16.0%)	48 (35.82%)	51 (38.06%)	35 (26.12%)	
BMI					0.85
Underweight (< 18)	30 (3.7%)	12 (40%)	12 (40%)	6 (20%)	
Normal (18 – 24.9)	503 (62.5%)	169 (33.6%)	215 (42.74%)	119 (23.66%)	
Overweight (25 – 29.9)	165 (20.5%)	55 (33.33%)	68 (41.21%)	42 (25.45%)	
Obese (30+)	107 (13.3%)	32 (29.91%)	43 (40.19%)	32 (29.91%)	
Cancer Stage					0.39
1	344 (41.2%)	125 (36.34%)	133 (38.66%)	86 (25%)	
2	435 (52.1%)	135 (31.03%)	194 (44.6%)	106 (24.37%)	
3	56 (6.7%)	17 (30.36%)	27 (48.21%)	12 (21.43%)	
Tumor Size					0.56
≤2 cm	500 (63.8%)	171 (34.2%)	206 (41.2%)	123 (24.6%)	
2-5 cm	246 (31.4%)	76 (30.89%)	113 (45.94%)	57 (23.17%)	
>5 cm	38 (4.9%)	10 (26.32%)	20 (52.63%)	8 (21.05%)	
Number of nodes					0.80
0	451 (54.1%)	156 (34.59%)	185 (41.02%)	110 (24.39%)	
1 – 3	255 (30.6%)	78 (30.59%)	111 (43.53%)	66 (25.88%)	
4 – 9	80 (9.6%)	25 (31.25%)	34 (42.5%)	21 (26.25%)	
10+	48 (5.8%)	17 (35.42%)	23 (47.92%)	8 (16.67%)	
Surgery type					0.17
Lumpectomy	421 (50.5%)	149 (35.39%)	165 (39.19%)	107 (25.42%)	
Mastectomy	413 (49.5%)	127 (30.75%)	188 (45.52%)	98 (23.73%)	
Estrogen/Progesterone Status					0.16
+/+	460 (56.8%)	161 (35%)	187 (40.65%)	112 (24.35%)	
+/-	80 (9.9%)	18 (22.5%)	35 (43.75%)	27 (33.75%)	
-/+	28 (3.5%)	10 (35.71%)	10 (35.71%)	8 (28.57%)	
-/-	242 (29.9%)	80 (33.06%)	112 (46.28%)	50 (20.66%)	
Histological Grade					0.53
Grade I	57 (8.2%)	21 (36.84%)	20 (35.09%)	16 (28.07%)	
Grade II	221 (31.9%)	73 (33.03%)	91 (41.18%)	57 (25.79%)	
Grade III	415 (59.9%)	131 (31.57%)	190 (45.78%)	94 (22.65%)	
Radiation therapy					0.60
No	490 (69.5%)	173 (35.31%)	197 (40.2%)	120 (24.49%)	
Yes	215 (30.5%)	72 (33.49%)	95 (44.19%)	48 (22.33%)	
Chemotherapy					0.0087
No	99 (11.8%)	46 (46.46%)	31 (31.31%)	22 (22.22%)	
Yes	737 (88.2%)	231 (31.34%)	323 (43.83%)	183 (24.83%)	
Hormone therapy					0.041
No	336 (40.4%)	119 (35.42%)	150 (44.64%)	67 (19.94%)	
Yes	496 (59.6%)	158 (31.85%)	201 (40.52%)	137 (27.62%)	
Hot flash					0.005
No	508 (60.8%)	189 (37.2%)	203 (40.0%)	116 (22.8%)	
Mild	159 (19.0%)	52 (32.7%)	72 (45.3%)	35 (22.0%)	
Moderate	121 (14.5%)	28 (23.1%)	58 (47.9%)	35 (28.9%)	
Severe	47 (5.6%)	8 (17.0%)	20 (42.6%)	19 (40.4%)	
Night Sweats					0.0006
No	494 (59.2%)	188 (38.1%)	199 (40.3%)	107 (21.7%)	
Mild	180 (21.6%)	55 (30.6%)	82 (45.6%)	43 (23.9%)	
Moderate	112 (13.4%)	28 (25%)	50 (44.6%)	34 (30.4%)	
Severe	49 (5.9%)	6 (12.2%)	22 (44.9%)	21 (42.9%)	
**SF-12 physical component score**	43.85 ± 9.07	46.38 ± 8.57	43.41 ± 9.23	41.22 ± 8.59	<0.0001
**SF-12 mental component score**	42.86 ± 8.33	44.48 ± 7.98	42.62 ± 8.36	41.12 ± 8.39	<0.0001

Bolded values are statistically significant.

Three distinct insomnia trajectories were identified: low probability of insomnia (n=277, 33.1%), moderate probability (n=354, 42.4%), and high probability (n=205, 24.5%) (**Figure 1**; **Table 2**). For those in the low and moderate probability trajectories, the estimated probability of insomnia was highest at baseline, declined during follow-up, and remained relatively low or moderate thereafter (**Figure 1**). However, those in the high probability group had no change in probability over time and remained persistently high. Average PPA ranged from 75% to 82% across trajectory groups. Average probability of insomnia was 5% in the low group, 43% in the moderate group, and 88% in the high group (**Table 2**). Missing insomnia symptoms assessments increased over follow-up, ranging across trajectory groups from 0%–1.1% at baseline, 5.4%–17.3% at 6 months, 8.8%–19.9% at 12 months, 17.6%–25.3% at 18 months, 22.0%–30.0% at 24 months, 50.2%–54.8% at 30 months, and 28.8%–35.9% at 36 months. Missingness differed significantly across trajectory groups at 6 and 12 months (p=0.0004 and p=0.03 respectively), with lower missingness in the high-probability insomnia group, but did not differ significantly across groups at the other assessments. In univariate models, those who were 40–45 years old had 2.47 times the odds of being in the high insomnia group vs. the low group compared to those who were <30 years old (95% CI: 1.10, 5.56; **Table 3**). Additionally, those with some college education (OR: 1.66, 95% CI: 1.04, 2.62), an income of <$50,000 (OR: 1.64, 95% CI: 1.02, 2.62), and other employment status (which included unemployed, retired, disabled, or other; OR: 1.65, 95% CI: 1.06, 2.58) had higher odds of being in the moderate or high probability insomnia group vs. the low probability insomnia group. In terms of clinical factors, those with ER-/PR+ cancer had 2.16 times the odds of being in the high group vs low group compared to those with ER+/PR+ cancer (95% CI: 1.13, 4.10). Also, those that received chemotherapy had over two times the odds of being in the moderate group vs low group (OR: 2.08, 95% CI: 1.28, 3.37) and those who received hormone therapy had 1.54 times the odds of being in the high group vs low group (95% CI: 1.06, 2.24). Participants who experienced moderate or severe hot flashes or night sweats also had much higher odds of being in the moderate and high groups vs the low group compared to those who did not have hot flashes or night sweats (ORs ranging from 1.93-6.15). Finally, higher SF-12 physical and mental component scores were associated with lower odds of being in the moderate and high groups vs low group (ORs ranging from 0.95-0.97).

**Figure 1:**
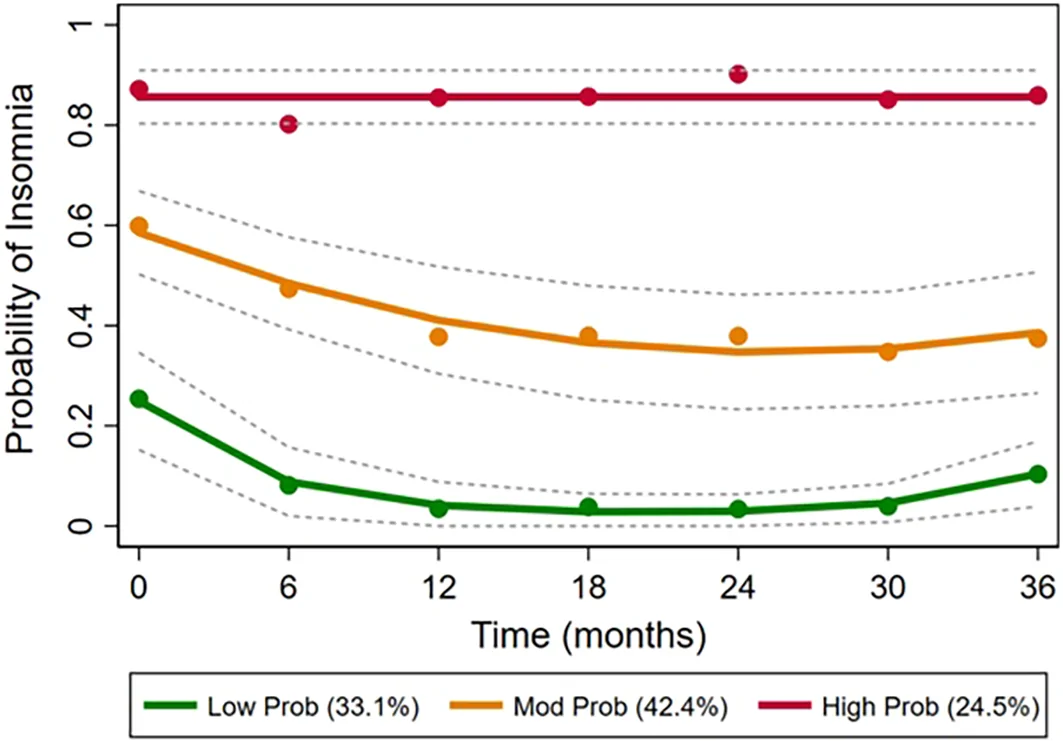
Three-group trajectory plots of WHIIRS scores over 36 months, with 95% confidence intervals.

**Table 2:** Insomnia trajectory group membership size and probability.

	Insomnia trajectory group		
	Low probability N=277 (33.1%)	Moderate probability N=354 (42.4%)	High probability N=205 (24.5%)
Probability of Group Membership (range: 0-1)	0.77	0.75	0.82
Mean WHIIRS score (range: 0-20)	3.86	7.90	12.61
Mean Probability of Insomnia (range: 0-1)	0.05	0.43	0.88

**Table 3:** Univariate models of the associations between various factors and moderate and high probability groups compared to low probability of insomnia.

	UNIVARIATE				
	Moderate vs Low		High vs Low		
** Characteristics**	OR (95% CI)	p-value	OR (95% CI)	p-value	Overall P-value
Age at Diagnosis					0.036
<30 years	ref		ref		
30 – 34.9 years	1.42 (0.72-2.80)	0.31	1.76 (0.72-4.27)	0.21	
35 – 39.9 years	1.24 (0.66-2.35)	0.5	1.44 (0.62-3.36)	0.40	
40 – 45 years	1.19 (0.64-2.21)	0.59	**2.47 (1.10-5.56)**	0.03	
Race					0.88
Non-Hispanic White	ref		ref		
Black/ African American	0.85 (0.43-1.71)	0.66	0.56 (0.23-1.40)	0.21	
Hispanic	0.76 (0.33-1.72)	0.51	0.75 (0.29-1.94)	0.55	
Asian/Pacific Islander	0.76 (0.3-1.94)	0.56	0.71 (0.24-2.17)	0.55	
Education					0.033
High school or less	0.90 (0.50-1.64)	0.73	0.79 (0.40-1.55)	0.49	
Some college	**1.66 (1.04-2.62)**	0.032	1.64 (0.99-2.70)	0.053	
College Degree	1.22 (0.83-1.79)	0.32	0.77 (0.49-1.20)	0.25	
Post-graduate	ref		ref		
Income					0.49
< $50,000	1.17 (0.78-1.75)	0.44	**1.64 (1.02-2.62)**	0.04	
$50,000-$74,999	0.96 (0.62-1.49)	0.84	1.44 (0.86-2.39)	0.16	
$75,000-$100,000	1.02 (0.64-1.64)	0.92	1.36 (0.79-2.35)	0.27	
> $100,000	ref		ref		
Employment					0.23
Full-time	ref		ref		
Part-time	1.48 (0.93-2.36)	0.10	0.95 (0.54-1.68)	0.86	
Other	**1.65 (1.06-2.58)**	0.028	1.50 (0.91-2.48)	0.11	
Homemaker	1.30 (0.84-2.00)	0.24	1.13 (0.69-1.86)	0.63	
Marital Status					0.77
Married/Partnered	ref		ref		
Divorced/Separated	1.25 (0.72-2.16)	0.43	1.13 (0.59-2.14)	0.72	
Never Married	0.82 (0.53-1.27)	0.38	0.99 (0.61-1.61)	0.98	
BMI					0.85
Underweight (< 18)	0.79 (0.34-1.79)	0.57	0.71 (0.26-1.95)	0.51	
Normal (18 – 24.9)	ref		ref		
Overweight (25 – 29.9)	0.97 (0.65-1.46)	0.89	1.08 (0.68-1.73)	0.73	
Obese (30+)	1.06 (0.64-1.74)	0.83	1.42 (0.83-2.45)	0.21	
Cancer Stage					0.39
1	ref		ref		
2	1.35 (0.97-1.88)	0.073	1.14 (0.79-1.66)	0.49	
3	1.49 (0.78-2.87)	0.23	1.03 (0.47-2.26)	0.95	
Tumor Size					0.56
≤2 cm	ref		ref		
2-5 cm	1.23 (0.87-1.76)	0.24	1.04 (0.69-1.58)	0.84	
>5 cm	1.66 (0.76-3.64)	0.21	1.11 (0.43-2.90)	0.93	
Number of nodes involved					0.80
0	ref		ref		
1 – 3	1.20 (0.84-1.72)	0.32	1.20 (0.80-1.81)	0.38	
4 – 9	1.15 (0.66-2.01)	0.63	1.19 (0.64-2.24)	0.59	
10+	1.14 (0.59-2.21)	0.70	0.67 (0.28-1.60)	0.37	
Surgery type					0.17
Lumpectomy	ref		ref		
Mastectomy	1.34 (0.98-1.83)	0.07	1.08 (0.75-1.54)	0.7	
Estrogen/Progesterone Status					0.17
+/+	ref		ref		
+/-	1.67 (0.91-3.07)	0.096	**2.16 (1.13-4.10)**	0.02	
-/+	0.86 (0.35-2.12)	0.74	1.15 (0.44-3.01)	0.78	
-/-	1.21 (0.84-1.72)	0.30	0.90 (0.59-1.38)	0.62	
Histological Grade					0.53
Grade I	ref		ref		
Grade II	1.31 (0.66-2.60)	0.44	1.02 (0.49-2.14)	0.95	
Grade III	1.52 (0.79-2.92)	0.21	0.94 (0.47-1.90)	0.87	
Radiation therapy					0.60
No	ref		ref		
Yes	1.16 (0.80-1.67)	0.43	0.96 (0.62-1.48)	0.86	
Chemotherapy					0.0098
No	ref		ref		
Yes	**2.08 (1.28-3.37)**	0.003	1.66 (0.96-2.85)	0.07	
Hormone therapy					0.042
No	ref		ref		
Yes	1.01 (0.73-1.39)	0.95	**1.54 (1.06-2.24)**	0.02	
Hot flash					0.0069
No	ref		ref		
Mild	1.29 (0.86-1.94)	0.22	1.10 (0.67-1.79)	0.71	
Moderate	**1.93 (1.18-3.16)**	0.009	**2.04 (1.18-3.52)**	0.01	
Severe	**2.33 (1.001-5.41)**	0.049	**3.87 (1.64-9.12)**	0.002	
Night Sweats					0.0013
No	ref		ref		
Mild	1.41 (0.95-2.09)	0.09	1.37 (0.86-2.19)	0.18	
Moderate	**1.69 (1.02-2.79)**	0.042	**2.13 (1.23-3.71)**	0.007	
Severe	**3.46 (1.37-8.73)**	0.008	**6.15 (2.41-15.71)**	0.0001	
**SF-12 physical component score#**	**0.96 (0.95-0.98)**	<.0001	**0.94 (0.92-0.96)**	<.0001	<.0001
**SF-12 mental component score#**	**0.97 (0.95-0.99)**	0.0053	**0.95 (0.93-0.97)**	<.0001	<.0001

#The SF-12 scores are out of 100 points, a higher score indicates better health, therefore an OR <1 shows lower odds of being in the moderate or high insomnia probability groups. Bolded values are statistically significant.

In the multivariable model, older age (40–45 years vs <30 years), education level (some college vs. post-graduate), receipt of chemotherapy, presence of night sweats, and lower SF-12 physical (poorer physical functioning) and mental component scores (worse mental health) remained significant factors associated with moderate and high probability of insomnia groups vs. low probability of insomnia group (**Table 4**). In sensitivity analyses accounting for uncertainty in trajectory classification using posterior-probability-based pseudo-class assignments, lower SF-12 physical and mental component scores remained significant factors associated with trajectory membership, whereas associations with age, education, chemotherapy, and night sweats were attenuated and no longer statistically significant (**Supplementary Table 2**).

**Table 4:** Multivariate models of the associations between various factors and moderate and high probability groups compared to low probability of insomnia.

	MULTIVARIATE				
	Moderate vs Low		High vs Low		
** Characteristics**	OR (95% CI)	p-value	OR (95% CI)	p-value	Overall P-value
Age at Diagnosis					0.16
<30 years	ref		ref		
30 – 34.9 years	1.63 (0.81-3.32)	0.17	2.14 (0.83-5.49)	0.11	
35 – 39.9 years	1.54 (0.79-2.99)	0.21	1.90 (0.78-4.66)	0.16	
40 - 45 years	1.49 (0.77-2.87)	0.23	**2.86 (1.20-6.82)**	**0.018**	
Education					0.083
High school or less	0.94 (0.50-1.78)	0.85	0.85 (0.40-1.81)	0.68	
Some college	**1.74 (1.07-2.82)**	**0.025**	**1.86 (1.07-3.20)**	**0.027**	
College Degree	1.25 (0.83-1.87)	0.29	0.90 (0.56-1.47)	0.68	
Post-graduate	ref		ref		
Radiation					0.46
No	ref		ref		
Yes	1.27 (0.86-1.89)	0.24	1.07 (0.66-1.73)	0.78	
Chemotherapy					0.102
No	ref		ref		
Yes	**1.78 (1.05-3.01)**	**0.033**	1.26 (0.68-1.35)	0.46	
Hormone therapy					0.17
No	ref		ref		
Yes	0.96 (0.68-1.35)	0.82	1.37 (0.91-2.08)	0.13	
Hot flash					0.65
No	ref		ref		
Mild	0.90 (0.52-1.57)	0.72	0.63 (0.33-1.23)	0.18	
Moderate	1.42 (0.66-3.09)	0.37	0.96 (0.39-2.36)	0.92	
Severe	0.66 (0.18-2.46)	0.53	0.70 (0.18-2.79)	0.61	
Night Sweats					0.36
No	ref		ref		
Mild	1.31 (0.77-2.25)	0.31	1.33 (0.71-2.48)	0.38	
Moderate	1.13 (0.51-2.48)	0.76	1.63 (0.66-4.02)	0.29	
Severe	3.78 (0.93-15.39)	0.064	**5.88 (1.33-25.92)**	**0.019**	
**SF-12 physical component score**	**0.95 (0.93-0.97)**	**0.0001**	**0.92 (0.89-0.94)**	**<0.0001**	<0.0001
**SF-12 mental component score**	**0.96 (0.94-0.98)**	**0.0004**	**0.92 (0.90-0.95)**	**<0.0001**	<0.0001

Bolded values are statistically significant.

## Discussion

We identified three distinct groups of insomnia trajectories over a 3-year period among young breast cancer survivors: low, moderate, and high probability of insomnia. Approximately, two-thirds of participants were in the moderate or high insomnia trajectories, indicating a substantial burden of persistent insomnia symptoms. Women on the moderate to high probability trajectories tended to be older (within this young cohort), reported more severe night sweats, and had poorer physical functioning and mental well-being. However, age and night sweats were attenuated in our sensitivity models. Overall, these findings suggest that insomnia symptoms present a chronic issue for a sizable number of young breast cancer survivors for many years post-diagnosis.

Our results extend prior longitudinal analyses of this cohort that examined average changes in insomnia symptoms over 3 years without differentiating distinct patterns (21). Consistent with that analysis, insomnia symptoms were most severe closer to diagnosis and improved over time on average. Yet a substantial proportion of women continued to meet WHIIRS criteria for high probability of clinically significant insomnia symptoms at 3 years. By applying group-based trajectory modeling in the current analyses we were able to identify a large subgroup of women whose probability of insomnia remained consistently high across the 3-year period and also determine both physical and psychological factors associated with their likelihood of insomnia. In a prior trajectory study using the WHIIRS among breast cancer patients, higher depressive symptoms, anxiety, and chronic life stress distinguished women in more severe insomnia trajectories, whereas oncologic factors showed weaker associations (32). Chemotherapy was more strongly related to trajectories of fatigue than insomnia itself, and chronic stressors were linked to persistent fatigue (32). Our findings are consistent with this pattern in that psychosocial and symptoms factors (e.g., physical and mental health, vasomotor symptoms) were more strongly associated with insomnia trajectory membership than tumor and treatment characteristics. Similarly, a 3-year study of sleep quality in breast cancer survivors identified low, medium, and high PSQI trajectories, with women in the low sleep quality trajectories more likely to have significant anxiety and depression at baseline and higher risk of neuropathic pain at 3 years (33). Together with our results, these findings suggest that high burden sleep trajectories co-occur with worse emotional health and pain, and may mark a broader chronic symptom phenotype in breast cancer survivorship (33).

A long-term follow-up of young breast cancer survivors over 15 years reported initial declines in health-related quality of life during treatment with improvement by 1–3 years, but also persistent elevations in insomnia, fatigue, and pain for many women over the longer term (34). The study concluded that supportive programs targeting sleep, fatigue, and pain may improve QoL in long term survivors, aligning with our finding that a substantial minority follow a high probability insomnia trajectory that need additional support to improve their sleep health (34). Other longitudinal research in breast cancer assessed sleep at five time points from pre-chemotherapy through 2 years post-chemotherapy (T1 pre-treatment, T2 mid-treatment, T3–6 month, T4–1 year, and T5–2 years post-treatment), and found that General Sleep Disturbance Scale (GSDS) scores were highest at mid-chemotherapy (T2 mean 48.9, SD 22.9) and gradually decreased over time, with clinically meaningful sleep disturbance observed during treatment that persisted into survivorship (35). Midnight and early morning awakening, and fatigue at work showed significant temporal change peaking at T2. Anxiety (T1, T2, and T4), fatigue (T3 and T4), and stress (T3) predicted sleep disturbance even after adjustment for radiation, surgery, and endocrine therapy (35). Our trajectory results complement these findings by showing that despite average recovery over time, a significant group of young survivors’ experience chronically high probability of insomnia.

A prospective study following women from before chemotherapy initiation, during (Cycle 4), and six months after chemotherapy initiation found persistently poor sleep quality at all timepoints, with strong associations between poor sleep, lower QoL, fatigue, depressive symptoms, and vasomotor symptoms (36). The study suggests early detection and intervention to minimize chronic insomnia during survivorship, which is directly supported by the high probability insomnia trajectory seen in our cohort. A common concern among breast cancer survivors is “chemo-brain,” referring to problems with memory, attention, and cognitive efficacy that can persist during and after chemotherapy. One potential contributor to these cognitive difficulties is disturbed sleep, because consolidated sleep plays a central role in encoding and consolidating new memories. Emerging work in breast cancer survivors shows that greater insomnia and sleep complaints are associated with poorer memory performance and greater perceived cognitive impairment, suggesting that persistent insomnia symptoms during and after treatment may exacerbate cancer-related cognitive problems and underscores the importance of protecting sleep quality during cancer treatment and survivorship (37).

### Strengths and limitations

Strengths of this study include the large sample size of younger women with a recent breast cancer diagnosis, repeated measures of a validated insomnia rating scale over 3 years, and extensive chart review of the participants’ clinical information relating to their breast cancer diagnosis. Our focus on women ≤45 years addresses an important gap, as most survivorship sleep studies involve older women or a broader age range.

Nevertheless, several limitations that should be considered. First, generalizability may be limited because this study included younger women, who were primarily non-Hispanic White, with early-stage breast cancer, who had regular menstrual cycles at diagnosis, as required by the parent study’s primary aims of menstrual cycle maintenance post-breast cancer treatment. Older women and more diverse cancer populations may experience different insomnia symptom trajectories. In addition, because participants were enrolled between 1998-2005, changes in cancer treatment, survivorship care, and management of sleep disturbances may limit the applicability of these findings to contemporary breast cancer populations and should be validated in more recently treated cohorts. Second, all potential factors associated with insomnia symptom trajectory membership were treated as time-stable. Many sociodemographics and clinical characteristics are not expected to vary over time, however, the psychosocial factors of physical and mental health functioning, and hot flashes or night sweats could have changed over time. The primary analysis was designed to evaluate baseline characteristics associated with subsequent trajectory membership. This approach does not evaluate whether changes in the potentially time-varying factors are associated with changes in insomnia symptoms; longitudinal models incorporating time-varying covariates would be needed to address that question. We also acknowledge insomnia symptoms and cancer-related symptoms may have bidirectional associations where symptoms such as night sweats or pain can contribute to sleep disturbances, while poor sleep may also exacerbate symptoms. Future studies using longitudinal time-varying analytic approaches are needed to better characterize the directionality of these relationships among young breast cancer survivors. Third, presence of insomnia symptoms was based on a self-reported measure and did not use objective sleep data or clinical diagnoses of insomnia. Actigraphy can be suboptimal in identifying insomnia, however, and self-reported perceptions of sleep disruption are a key part of reporting insomnia symptoms. The WHIIRS is a validated, widely used measure of insomnia symptoms (28). Finally, attrition increased over follow-up and was substantial at some assessments. Although missingness did not differ across trajectory groups at most time points, participants in the high-probability insomnia trajectory had significantly less missing data at 6 and 12 months. Thus, differential missingness and nonrandom attrition may have influenced trajectory estimation and group assignment.

## Conclusions

Physical and mental health were strongly associated with insomnia trajectory membership over three years among young breast cancer survivors. Identifying trajectories of insomnia symptoms may help inform future efforts aimed at developing and testing targeted intervention strategies. Future studies are needed to determine whether early identification and intervention can modify these insomnia symptom trajectories and improve broader survivorship outcomes.

## Data Availability

The data analyzed in this study is subject to the following licenses/restrictions: The de-identified data that support the findings of this study are available from the senior author, M.N., upon reasonable request. Submission of a short proposal for data use and approval are required. Requests to access these datasets should be directed to Michelle.Naughton@osumc.edu.
