# Supplementary material for: Three-year trajectories of insomnia symptoms among young breast cancer survivors
Source: Front Oncol. 2026 Oct 19;16:1967396. doi: 10.3389/fonc.2026.1967396 (PMC13724340; doi:10.3389/fonc.2026.1967396)
Supplement: Supplementary file 1 [file Table1.docx]

Supplemental Table 1. Model fit and classification characteristics of candidate group-based trajectory models for insomnia.

| No. groups | Polynomial order | BIC (N=4475) | AIC | Entropy | Assigned Class sizes, n(%) | Mean PPA by class |
| --- | --- | --- | --- | --- | --- | --- |
| 2 | 0, 0 | -2735.17 | -2725.56 | 0.67 | 510 (61.0%); 326 (39.0%) | 0.89; 0.92 |
| 3 | 0, 0, 0 | -2791.58 | -2703.56 | 0.55 | 294 (35.2%); 339 (40.6%); 203 (24.3%) | 0.78; 0.77; 0.78 |
| 4 | 0, 0, 0 ,0 | -2727.98 | -2705.56 | 0.49 | 244 (29.2%); 389 (46.5%); 203 (24.3%); 0 (0%) | 0.49; 0.73, 0.78; --- |
| 2 | 1, 1 | -2713.30 | -2697.29 | 0.66 | 500 (59.8%); 336 (40.2%) | 0.91; 0.88 |
| 3 | 1, 1, 1 | -2692.66 | -2667.04 | 0.61 | 182 (21.8%); 387 (46.3%); 267 (31.9%) | 0.77; 0.83; 0.80 |
| 4 | 1, 1, 1, 1 | -2694.38 | -2659.15 | 0.61 | 69 (8.2%); 400 (47.9%); 195 (23.3%); 172 (20.6%) | 0.73; 0.73; 0.82; 0.78 |
| 3 | 1, 1, 0 | -2688.55 | -2666.13 | 0.61 | 182 (21.8%); 387 (46.3%); 267 (31.9%) | 0.77; 0.83; 0.80 |
| 3 | 2, 2, 1 | -2682.46 | -2650.43 | 0.56 | 281 (33.6%); 362 (43.3%); 193 (23.1%) | 0.79; 0.75; 0.82 |
| **3** | **2, 2, 0** | **-2678.90** | **-2650.07** | **0.55** | **277 (33.1%); 354 (42.4%); 205 (24.5%)** | **0.77, 0.75, 0.82** |
| 3 | 3, 3, 0 | -2685.87 | -2650.64 | 0.55 | 281 (33.6%); 362 (43.3%); 193 (23.1%) | 0.78, 0.75, 0.82 |
| 4 | 2, 2, 0, 0 | -2682.00 | -2646.76 | 0.52 | 296 (35.4%); 137 (16.4%); 157 (18.8%); 246 (29.4%) | 0.68, 0.70, 0.77, 0.76 |

**Note:** The final selected three-group trajectory model is shown in bold.

Supplemental Table 2: Sensitivity analysis of multivariable associations with insomnia trajectory membership accounting for classification uncertainty.

|  | **Moderate vs Low** |  | **High vs Low** |  |
| --- | --- | --- | --- | --- |
| **Characteristics** | OR (95% CI) | p-value | OR (95% CI) | p-value |
| **Age at Diagnosis** |  |  |  |  |
| <30 years | ref |  | ref |  |
| 30 – 34.9 years | 1.24 (0.47-3.28) | 0.66 | 1.64 (0.48-5.56) | 0.43 |
| 35 – 39.9 years | 1.46 (0.58-3.69) | 0.42 | 1.84 (0.58-5.90) | 0.30 |
| 40 - 45 years | 1.36 (0.55-3.36) | 0.50 | 2.22 (0.72-6.84) | 0.16 |
| **Education** |  |  |  |  |
| High school or less | 1.13 (0.47-2.74) | 0.78 | 1.07 (0.40-2.89) | 0.89 |
| Some college | 1.46 (0.77-2.75) | 0.25 | 1.77 (0.89-3.53) | 0.10 |
| College Degree | 0.98 (0.56-1.71) | 0.93 | 0.81 (0.43-1.53) | 0.52 |
| Post-graduate | ref |  | ref |  |
| **Radiation** |  |  |  |  |
| No | ref |  | ref |  |
| Yes | 1.18 (0.71-1.96) | 0.52 | 1.03 (0.58-1.81) | 0.92 |
| **Chemotherapy** |  |  |  |  |
| No | ref |  | ref |  |
| Yes | 1.63 (0.81-3.25) | 0.17 | 1.31 (0.59-2.90) | 0.50 |
| **Hormone therapy** |  |  |  |  |
| No | ref |  | ref |  |
| Yes | 0.97 (0.60-1.55) | 0.89 | 1.26 (0.74-2.14) | 0.39 |
| **Hot flash** |  |  |  |  |
| No | ref |  | ref |  |
| Mild | 0.86 (0.41-1.81) | 0.69 | 0.61 (0.27-1.38) | 0.24 |
| Moderate | 1.06 (0.38-3.00) | 0.91 | 1.24 (0.42-3.70) | 0.70 |
| Severe | 0.68 (0.11-4.10) | 0.67 | 0.60 (0.10-3.54) | 0.57 |
| **Night Sweats** |  |  |  |  |
| No | ref |  | ref |  |
| Mild | 1.20 (0.58-2.47) | 0.62 | 1.43 (0.66-3.11) | 0.37 |
| Moderate | 1.23 (0.43-3.50) | 0.70 | 1.44 (0.46-4.53) | 0.53 |
| Severe | 2.68 (0.41-17.42) | 0.30 | 5.21 (0.81-33.53) | 0.082 |
| **SF-12 physical component score** | **0.96 (0.94-0.99)** | **0.01** | **0.93 (0.90-0.95)** | **<0.0001** |
| **SF-12 mental component score** | **0.96 (0.93-0.99)** | **0.0047** | **0.93 (0.90-0.96)** | **<0.0001** |
